# Ophthalmomyiasis Outbreak Caused by *Oestrus ovis* Infection, Algeria, 2025

**DOI:** 10.3201/eid3207.260552

**Published:** 2026-07

**Authors:** Yuyang Zeng, Hongkuan Yang, Xin Li, Hongzheng Yang, Yunyun Zhou

**Affiliations:** Spencer Center for Vision Research Department of Ophthalmology, Byers Eye Institute at Stanford University School of Medicine, Palo Alto, California, USA (Y. Zeng); Renmin Hospital of Wuhan University Department of Ophthalmology, Wuhan, China (Y. Zeng, Y. Zhou); Tongji Hospital of Huazhong University of Science and Technology Department of Neurosurgery, Wuhan (Hongkuan Yang); Hubei Cancer Hospital of Huazhong University of Science and Technology Department of Anesthesiology, Wuhan (X. Li); Renhe Hospital Affiliated with China Three Gorges University Department of Medical Cosmetology, Yichang, China (Hongzheng Yang)

**Keywords:** Ophthalmology, parasites, ophthalmomyiasis, *Oestrus ovis*, sheep, zoonoses, Algeria

## Abstract

Ophthalmomyiasis is a rare eye infestation caused by fly larvae and more often seen in rural areas. We report an outbreak of 17 patients in Algeria with ophthalmomyiasis after sheep exposure. All patients fully recovered after removal of ocular *Oestrus ovis* larvae and topical therapy, highlighting the effectiveness of early detection and treatment.

Ophthalmomyiasis is a rare ocular infestation in modern clinical settings. *Oestrus ovis*, the sheep nasal bot fly, is the most common cause of human cases (*1*). Because *O. ovis* larvae primarily infect sheep and goats, human infection occurs predominantly in rural settings, although urban cases have been reported (*2*–*4*). Ophthalmomyiasis is classified as external, internal, or orbital, on the basis of infestation site. External ophthalmomyiasis is limited to the ocular surface, involving the conjunctiva and cornea (*5*). Internal ophthalmomyiasis affects intraocular structures including the anterior chamber, choroid, and vitreous (*6*). Last, orbital ophthalmomyiasis involves the orbital cavity and adjacent tissues. Larval migration and intraocular involvement can cause structural damage and vision loss (*7*).

We report a case series of 17 patients with acute external ophthalmomyiasis caused by *O. ovis* infection after sheep exposure during ritual sacrifice for Eid al-Adha in Algeria during June 6–8, 2025. Patients were 26–45 years of age; 10 were men and 7 were women. Sixteen patients reported exposure during Eid al-Adha, whereas 1 patient denied direct or indirect sheep exposure but suspected a foreign body in the eye. The time from exposure to hospital visit ranged from 5 to 32 hours, and symptom onset occurred 1–10 hours after exposure (Appendix Table, https://wwwnc.cdc.gov/EID/article/32/7/26-0552-App1.pdf).

Clinical manifestations included palpebral edema, conjunctival hyperemia, pruritus, foreign body sensation, epiphora, chemosis, photophobia, and pricking pain (Figure 1, panels A–C). All cases were unilateral. Slit-lamp examination revealed numerous and motile larvae on the cornea, bulbar conjunctiva, and upper and lower conjunctival fornices with active movement (Figure 1, panels D and E; Video). Conjunctivitis-like signs included conjunctival congestion or edema, mucous discharge, and punctate keratitis (Figure 1, panels F–I). Corneal impairment was observed in 10 of 17 cases, including punctate keratitis in most and epithelial defects in 3 severe cases. Larvae measured ≈1–2 mm (Figure 2, panels A, B), and 4–22 larvae were identified per affected eye. Intraocular pressure was normal in all patients. Funduscopic examination and optical coherence tomography did not reveal posterior segment abnormalities (Appendix Figure).

**Figure 1:**
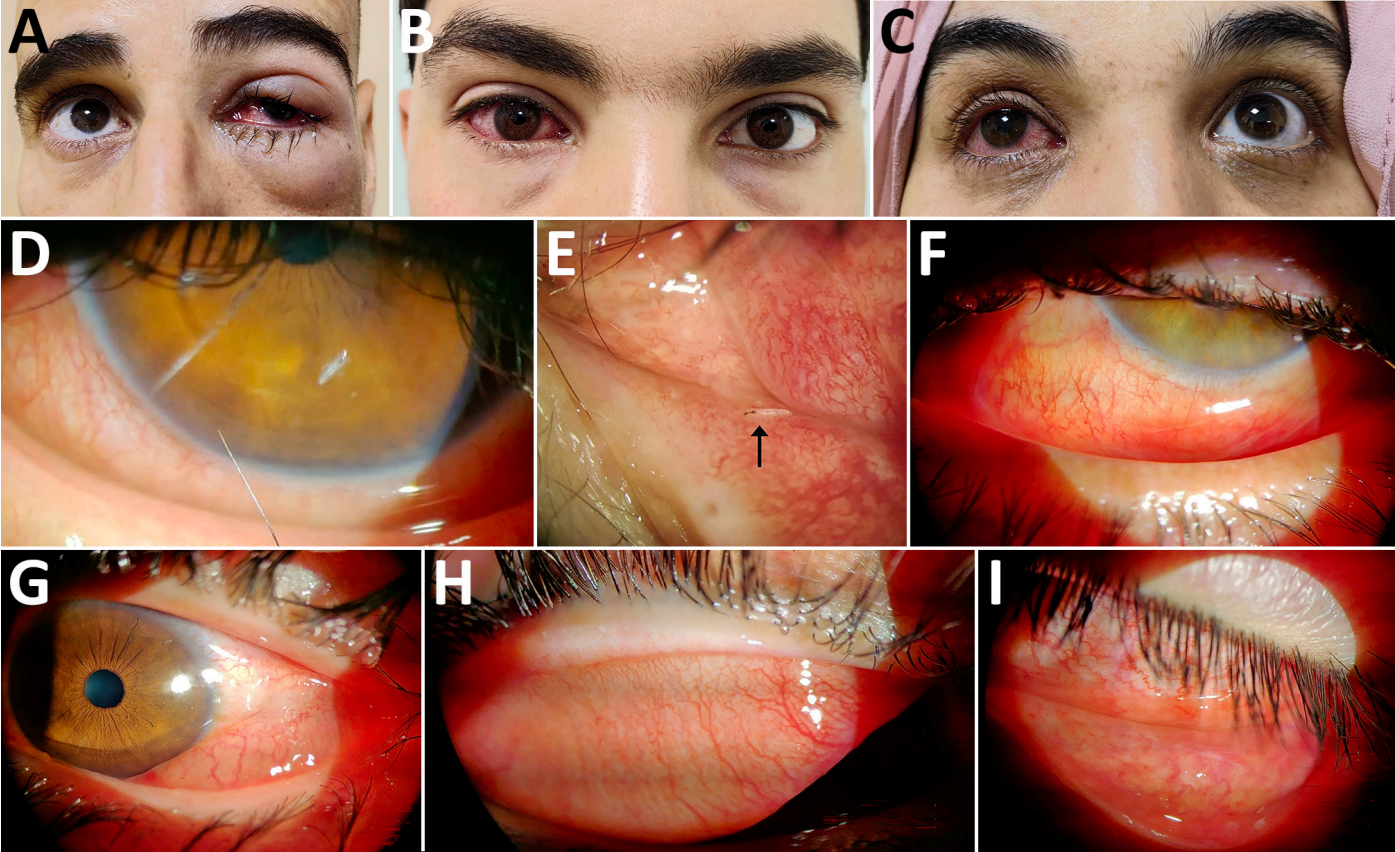
Clinical ocular findings in patients with acute external ophthalmomyiasis caused by *Oestrus ovis* infection after sheep exposure during Eid al-Adha, Algeria, 2025. A–C) Representative external ocular photographs show acute conjunctivitis-like findings, including palpebral edema, conjunctival hyperemia, and mucous discharge. D, E) Slit-lamp examination images show motile larvae on the corneal surface and in the lower conjunctival fornix. F–I) Slit-lamp examination images show conjunctival inflammation, including conjunctival congestion, edema, and mucous discharge. Black arrow in panel E indicates the location of an *O. ovis* larva.

**Video:** Representative video showing *Oestrus ovis* larvae in patient with acute external ophthalmomyiasis caused by *Oestrus ovis* infection after sheep exposure during Eid al-Adha, Algeria, 2025. Larvae exhibit rapid, active motility and wriggling along the surface of the inferior conjunctival fornix and on the corneal surface. The smooth corneal epithelium appeared to reduce their locomotor velocity.

**Figure 2:**
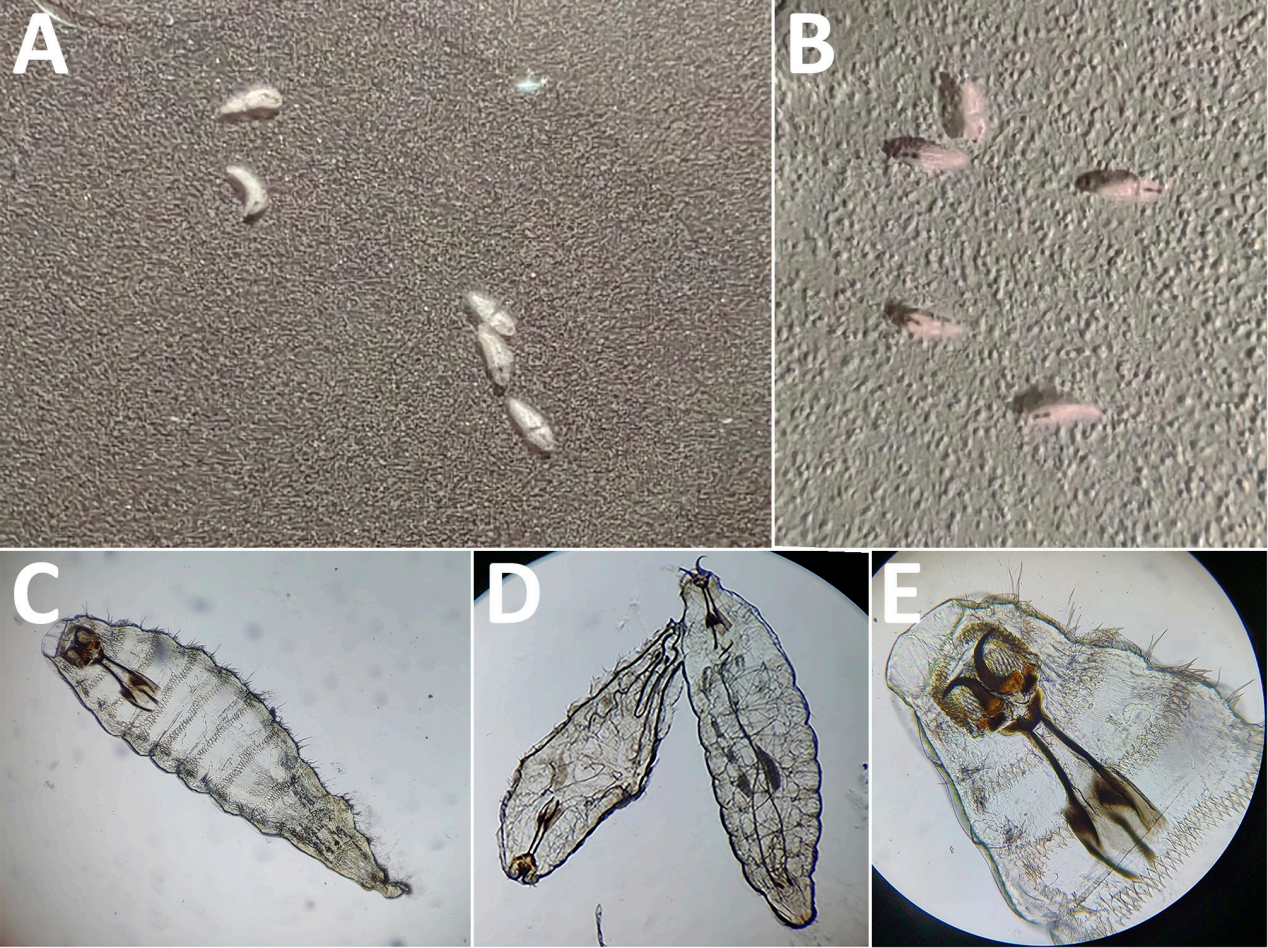
Morphologic identification of *Oestrus ovis* larvae extracted from patients with acute external ophthalmomyiasis after sheep exposure during Eid al-Adha, Algeria, 2025. A, B) Extracted first-instar larva from the ocular surface is shown grossly. C–E) Microscopic examination demonstrates characteristic features of *O. ovis*, including prominent oral hooks connected to the internal cephalopharyngeal skeleton and multiple rows of spiny projections. Original magnification ×40 (panel C), ×10 (panel D), and ×100 (panel E).

We rinsed and immersed extracted specimens in phosphate-buffered saline before submission for parasitologic analysis at the Parasitology Laboratory, Tongji Medical College, Huazhong University of Science and Technology (Wuhan, China). We treated the specimens with lactic acid–phenol, and microscopic examination revealed internal larval structures that included body segments, spines, spicules, cephalic oral hooks, valves (shape and number of stomata), and the cephalopharyngeal skeleton, consistent with *O. ovis* larvae (Figure 2, panels C–E).

We removed all visible larvae from infected patients and subsequently treated the patients with topical antimicrobial drug eye drops (4×/d for 1 wk) and neomycin/polymyxin B/dexamethasone ophthalmic ointment (1×/d for 1 wk), except in patients with corneal lesions. All patients achieved complete clinical resolution within 1–2 weeks without complications.

From our investigation, we believe that *O. ovis* larvae entered the patients’ eyes when adult flies deposited first-stage larvae. The substantial larval burden contributed to conjunctival inflammation and superficial corneal abrasions through cephalic oral hooks and body spicules.

*O. ovis* ophthalmomyiasis is traditionally associated with sheep- and goat-rearing areas in warm, dry Mediterranean climates (*8*). However, recent European reports (*2*–*4*) suggest broader geographic distribution; cases have been described in temperate or urban areas (*9*) and sometimes without clear livestock exposure. External ophthalmomyiasis might be overlooked because it closely mimics acute viral or bacterial conjunctivitis or nonspecific inflammatory ocular irritation (*1*,*10*). Thorough ocular examination and detailed livestock exposure history are therefore essential for timely diagnosis. Prompt mechanical removal of all larvae is critical to prevent intraocular involvement. This large cluster of 17 patients with ophthalmomyiasis underscores the potential for zoonotic ocular infestation from unprotected sheep contact during seasonal religious rituals. Public education, eye protection, and hygiene practices during ritual slaughter might help reduce similar outbreaks in endemic regions.

Appendix Additional information about ophthalmomyiasis outbreak caused by *Oestrus ovis* infection, Algeria, 2025.
