# Supplementary figures and images for: Ophthalmomyiasis Outbreak Caused by Oestrus ovis Infection, Algeria, 2025
Source: Emerg Infect Dis. 2026 Jul;32(7):1201–4. doi: 10.3201/eid3207.260552 (PMC13322440; doi:10.3201/eid3207.260552)

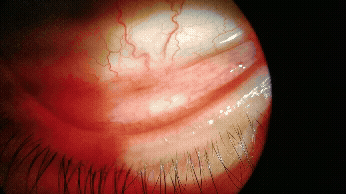

Supplement: Supplementary file 1 [file 26-0552-V.gif]
