# Supplementary material for: Ophthalmomyiasis Outbreak Caused by Oestrus ovis Infection, Algeria, 2025
Source: Emerg Infect Dis. 2026 Jul;32(7):1201–4. doi: 10.3201/eid3207.260552 (PMC13322440; doi:10.3201/eid3207.260552)
Supplement: Appendix — Additional information about ophthalmomyiasis outbreak caused by Oestrus ovis infection, Algeria, 2025. [file 26-0552-Techapp-s1.pdf]

Article DOI: https://doi.org/10.3201/eid3207.260552

*EID cannot ensure accessibility for supplementary materials supplied by authors. Readers who have difficulty accessing supplementary content should contact the authors for assistance.*

# Ophthalmomyiasis Outbreak Caused by *Oestrus ovis* Infection, Algeria, 2025

## Appendix

**Appendix Table 1.** Demographic characteristics, sheep exposure history, ocular findings, larvae burden, and timing of disease onset among 17 patients with acute external ophthalmomyiasis caused by *Oestrus ovis* after sheep exposure during Eid al-Adha, Algeria, 2025*

| Patient No. | Sex | Age | Affected eye | Sheep exposure | Symptoms | Slit-lamp examination findings | Larvae numbers | From exposure to first symptom | From exposure to slit-lamp examination |
|---|---|---|---|---|---|---|---|---|---|
| 1 | F | 35 | OS | Yes | Foreign body sensation, redness, itching, tearing, Feeling of fullness, pricking pain, photophobia | motile larvae (1–2mm) in conjunctiva, palpebral edema, conjunctival congestion, mucous discharge, epiphora, mucous discharge, punctate keratitis | 22 | 2h | 5h |
| 2 | F | 26 | OD | Yes | Foreign body sensation, redness | motile larvae (1–2mm) in conjunctiva, conjunctival congestion, mucous discharge | 4 | 4h | 6h |
| 3 | M | 46 | OS | Yes | Foreign body sensation, redness, Feeling of fullness, eye discharge | motile larvae (1–2mm) in conjunctiva, conjunctival congestion, edema, mucous discharge | 7 | 6h | 10h |
| 4 | M | 28 | OS | Yes | Foreign body sensation, redness, itching, tearing, Feeling of fullness, pricking pain, photophobia, eye discharge, burning feeling | motile larvae (1–2mm) in conjunctiva, conjunctival congestion, edema, mucous discharge, epiphora, corneal epithelial defects | 15 | 4h | 9h |
| 5 | M | 37 | OS | Yes | Foreign body sensation, redness, itching, tearing, Feeling of fullness, pricking pain, photophobia, eye discharge | motile larvae (1–2mm) in conjunctiva, conjunctival congestion, edema, mucous discharge, epiphora, punctate keratitis | 18 | 1h | 8h |
| 6 | F | 38 | OS | Yes | Foreign body sensation, redness, itching, tearing, feeling of fullness, pricking pain, photophobia, eye discharge | motile larvae (1–2mm) in conjunctiva, conjunctival congestion, edema, mucous discharge, epiphora, punctate keratitis | 16 | 2h | 7h |
| 7 | F | 42 | OS | Yes | Foreign body sensation, redness, pricking pain, burning feeling, eye discharge | motile larvae (1–2mm) in conjunctiva, conjunctival congestion, mucous discharge | 8 | 5h | 8h |

| Patient No. | Sex | Age | Affected eye | Sheep exposure | Symptoms | Slit-lamp examination findings | Larvae numbers | From exposure to first symptom | From exposure to slit-lamp examination |
|---|---|---|---|---|---|---|---|---|---|
| 8 | M | 34 | OD | Yes | Foreign body sensation, redness, itching, tearing, pricking pain, feeling of fullness, photophobia, eye discharge | motile larvae (1–2mm) in conjunctiva, conjunctival congestion, edema, mucous discharge, punctate keratitis | 14 | 1h | 9h |
| 9 | M | 43 | OS | Yes | Foreign body sensation, redness, eye discharge | motile larvae (1–2mm) in conjunctiva, conjunctival congestion, mucous discharge | 7 | 5h | 10h |
| 10 | M | 39 | OS | Yes | Foreign body sensation, redness, itching, tearing, feeling of fullness, pricking pain, photophobia, burning feeling, eye discharge | motile larvae (1–2mm) in conjunctiva, conjunctival congestion, edema, mucous discharge, corneal epithelial defects | 17 | 3h | 25h |
| 11 | M | 45 | OS | Yes | Foreign body sensation, redness, itching, tearing, feeling of fullness, pricking pain, photophobia, burning feeling, eye discharge | motile larvae (1–2mm) in conjunctiva, conjunctival congestion, edema, epiphora, mucous discharge, corneal epithelial defects | 9 | 4h | 29h |
| 12 | M | 41 | OD | Yes | Foreign body sensation, redness, tearing, pricking pain, photophobia, burning feeling, eye discharge | motile larvae (1–2mm) in conjunctiva, conjunctival congestion, edema, epiphora, mucous discharge, punctate keratitis | 5 | 3h | 27h |
| 13 | F | 32 | OD | Yes | Foreign body sensation, redness, itching, pricking pain | motile larvae (1–2mm) in conjunctiva, conjunctival congestion, punctate keratitis | 7 | 5h | 26h |
| 14 | F | 33 | OS | Yes | Foreign body sensation, redness, itching, pricking pain, feeling of fullness, eye discharge | motile larvae (1–2mm) in conjunctiva, conjunctival congestion, edema, mucous discharge, punctate keratitis | 6 | 2h | 32h |
| 15 | M | 36 | OD | Yes | Foreign body sensation, redness, itching, feeling of fullness, photophobia, burning feeling, eye discharge | motile larvae (1–2mm) in conjunctiva, conjunctival congestion, edema, mucous discharge | 9 | 4h | 30h |
| 16 | F | 41 | OS | Yes | Foreign body sensation, redness, itching, feeling of fullness, eye discharge | motile larvae (1–2mm) in conjunctiva, conjunctival congestion, edema, mucous discharge | 8 | 10h | 28h |
| 17 | M | 44 | OS | No | Foreign body sensation, redness, itching | motile larvae (1–2mm) in conjunctiva, conjunctival congestion, edema | 7 | - | 0h |

*F indicates female; M, male; OD, right eye; OS, left eye; h, hours; and mm, millimeters. Larvae were clinically identified as *Oestrus ovis*. The final 2 columns indicate the intervals from sheep exposure to first symptom onset and to slit-lamp examination, respectively.

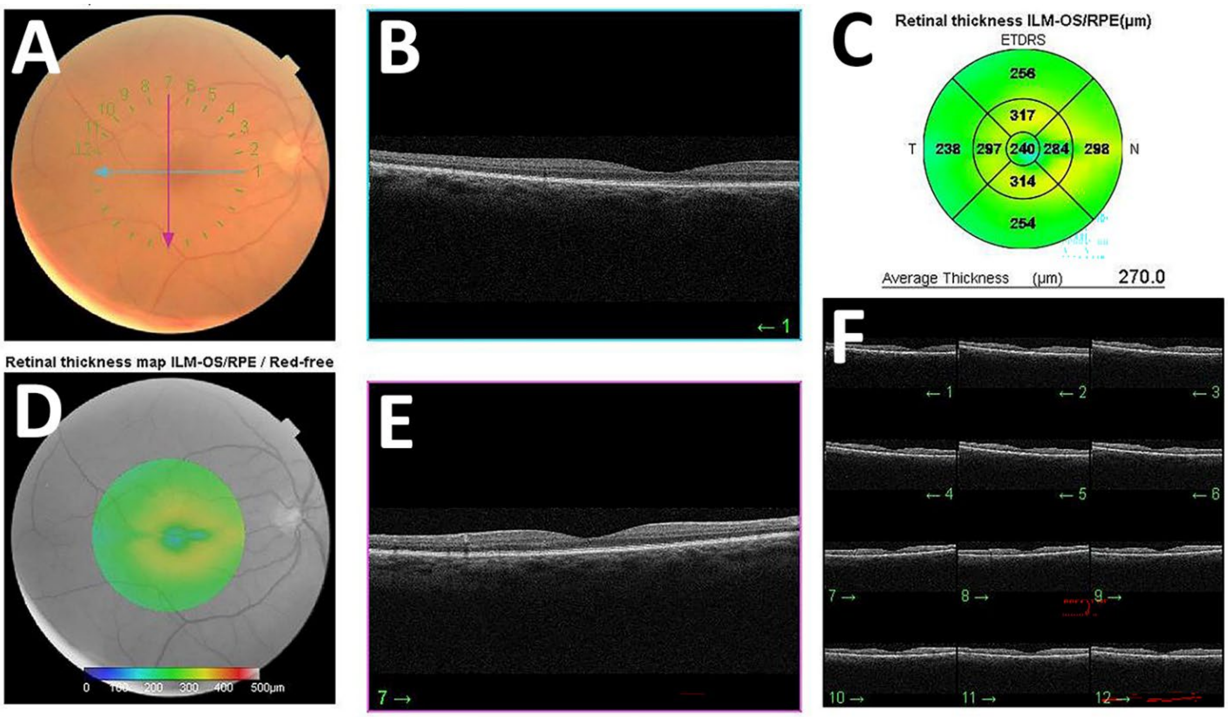


**Appendix Figure.** Posterior segment evaluation in patients with acute external ophthalmomyiasis caused by *Oestrus ovis* after sheep exposure during Eid al-Adha, Algeria, 2025. Representative color fundus photography, red-free fundus imaging, and optical coherence tomography images show no evidence of posterior segment involvement, including no retinal, choroidal, vitreous, or optic nerve abnormalities attributable to intraocular larval migration. OCT, optical coherence tomography.
